# A Patient-Derived Xenograft Model of Dedifferentiated Endometrial Carcinoma: A Proof-of-Concept Study for the Identification of New Molecularly Informed Treatment Approaches

**DOI:** 10.3390/cancers13235962

**Published:** 2021-11-26

**Authors:** Chiao-Yun Lin, Ren-Chin Wu, Chen-Yang Huang, Chyong-Huey Lai, An-Shine Chao, Hsin-Pai Li, Chia-Lung Tsai, Elizabeth Joo-Wen Kuek, Cheng-Lung Hsu, Angel Chao

**Affiliations:** 1 Department of Obstetrics and Gynecology, Linkou Chang Gung Memorial Hospital and College of Medicine, Chang Gung University, Taoyuan 333, Taiwan; chiao.yun0101@gmail.com (C.-Y.L.); laich46@cgmh.org.tw (C.-H.L.); aschao1295@cgmh.org.tw (A.-S.C.); 2 Gynecologic Cancer Research Center, Linkou Chang Gung Memorial Hospital, Taoyuan 333, Taiwan; renchin.wu@gmail.com; 3 Department of Pathology, Linkou Chang Gung Memorial Hospital and Chang Gung University College of Medicine, Taoyuan 333, Taiwan; 4 Division of Hematology-Oncology, Department of Internal Medicine, Linkou Chang Gung Memorial Hospital and Chang Gung University, Taoyuan 333, Taiwan; b9202070@cgmh.org.tw (C.-Y.H.); elizabethwen94@gmail.com (E.J.-W.K.); 5 New Taipei Municipal Tu Cheng Hospital, New Taipei City 236, Taiwan; 6 Molecular Medicine, Research Center, Department of Microbiology and Immunology, Chang Gung University, Taoyuan 333, Taiwan; paili@mail.cgu.edu.tw; 7 Genome Medicine Core Laboratory, Linkou Chang Gung Memorial Hospital, Taoyuan 333, Taiwan; cltsai24@gmail.com; 8 School of Medicine, Chang Gung University, Taoyuan 333, Taiwan

**Keywords:** dedifferentiated endometrial carcinoma, patient-derived xenograft models, targeted treatment, proof-of-concept, lenvatinib, palbociclib

## Abstract

**Simple Summary:**

Reliable animal models of human malignancies are paramount for preclinical studies of novel treatment approaches. Here, we successfully developed a patient-derived xenograft (PDX) model of dedifferentiated endometrial carcinoma (DEC)–an uncommon uterine malignancy that is generally unresponsive to standard chemo- and radiotherapy. The murine model–termed PDX-mLung–was established through the implantation of lung metastatic lesions obtained from a woman with DEC. Histologic and molecular findings revealed that PDX-mLung was highly similar to the parent human malignant lesions (both primary DEC and lung metastases). Importantly, molecular analyses revealed that PDX-mLung exhibited druggable alterations including a *FGFR2* mutation and *CCNE2* amplification. The former was targeted with the FGFR inhibitor lenvatinib while the latter with the cell cycle inhibitor palbociclib. The combination of the two drugs exhibited synergistic therapeutic effects against in vivo tumor growth. Collectively, these data illustrate the value of PDX models for preclinical testing of new molecularly informed therapies in difficult-to-treat gynecologic malignancies. Our results may also prompt further clinical research to examine whether the combination of lenvatinib and palbociclib has potential to improve clinical outcomes of women with DEC.

**Abstract:**

Conventional treatment of dedifferentiated endometrial carcinoma (DEC)–an uncommon and highly aggressive uterine malignancy–is beset by high failure rates. A line of research that holds promise to overcome these limitations is tailored treatments targeted on specific molecular alterations. However, suitable preclinical platforms to allow a reliable implementation of this approach are still lacking. Here, we developed a patient-derived xenograft (PDX) model for preclinical testing of investigational drugs informed by molecular data. The model–termed PDX-mLung was established in mice implanted with lung metastatic lesions obtained from a patient with DEC. Histologic and whole-exome genetic analyses revealed a high degree of identity between PDX-mLung and the patient’s parental lesions (both primary DEC and lung metastases). Interestingly, molecular analyses revealed that PDX-mLung harbored druggable alterations including a *FGFR2* mutation and *CCNE2* amplification. Targeted combined treatment with the FGFR inhibitor lenvatinib and the cell cycle inhibitor palbociclib was found to exert synergistic therapeutic effects against in vivo tumor growth. Based on the results of RNA sequencing, lenvatinib and palbociclib were found to exert anti-tumor effects by interfering interferon signaling and activating hormonal pathways, respectively. Collectively, these data provide proof-of-concept evidence on the value of PDX models for preclinical testing of molecularly informed drug therapy in difficult-to-treat human malignancies. Further clinical research is needed to examine more rigorously the potential usefulness of the lenvatinib and palbociclib combination in patients with DEC.

## 1. Introduction

Despite decades of medical research for what remains a significant public health problem in women, the incidence of endometrial cancer (EC) has been growing steadily [1,2]. While cytoreductive surgery remains the mainstay of therapy, chemo- and radiotherapy have clinical applications in patients with late-stage tumors [3]. Dedifferentiated endometrial carcinoma (DEC)–an uncommon and highly aggressive subtype of EC–is histologically characterized by mixed low-grade and undifferentiated endometrioid carcinoma components [4]. The clinical management of patients with DEC continues to be faced by undeniable challenges, and current multimodal treatment attempts are beset by high failure rates [4]. In general, DEC is not amenable to either hormone therapy or targeted cancer therapy owing to its minimal or absent expression of estrogen receptors (ER), progesterone receptors (PR), cytokeratins, and PAX8 [5,6,7]. They are involved in chromatin remodeling through SWI/SNF with the loss of BRG-1 (protein of *SMARCA4*), INI-1 (protein of *SMARCB1*), and ARID1A [5,6,7].

In this scenario, the development of patient-derived xenograft (PDX) models for preclinical testing of investigational drugs is a line of research that holds promise to address the large unmet need for effective interventions. Although not yet implemented on a large scale, PDX models are thought to provide a valuable platform for preclinical pharmacological research by retaining most properties of the cancer of origin–including histology and molecular alterations [8,9,10,11,12]. While PDX models of EC have been previously applied for preclinical studies and biomarker investigations of treatment response, the results were not thoroughly informed by the tumor’s molecular underpinnings. In this regard, a promising path that may work to expand the current therapeutic armamentarium in DEC is a more precise drug targeting based on the underlying molecular alterations.

Starting from these premises, we devised the current study with three main goals. First, we sought to establish a PDX model that could serve as a reliable preclinical platform by thoroughly reflecting the key histologic and molecular features of human DEC. Second, we undertook a detailed molecular characterization of the experimental tumor with the ultimate goal of identifying druggable alterations. Finally, we used the PDX model to conduct a proof-of-concept preclinical study aimed at assessing whether pharmacological targeting of specific molecular derangements would significantly inhibit tumor growth.

## 2. Materials and Methods

### 2.1. Collection of Specimens from a Woman with Dedifferentiated Endometrial Carcinoma

A 48-year-old woman who presented with abnormal uterine bleeding underwent diagnostic curettage and was originally diagnosed with moderately differentiated endometrioid adenocarcinoma based on biopsy results. The results of needle aspiration of a left supraclavicular lymph node revealed distant metastasis. She subsequently underwent laparotomic hysterectomy accompanied by pelvic washing, bilateral salpingo-oophorectomy, pelvic and paraaortic lymphadenectomy, and left supraclavicular lymph node dissection. The final pathological diagnosis on surgical specimens revealed the presence of DEC, composed mainly of undifferentiated carcinoma cells with a minor (<1%) focus of well differentiated endometrioid carcinoma. On immunohistochemistry (IHC), undifferentiated carcinoma cells were negative for AE1/AE3, CAM5.2, ER, and PR, and showed no loss of expression for INI1, BRG-1, and DNA mismatch-repair proteins (MLH1, PMS2, MSH2, and MSH6). Wildtype p53 staining pattern was observed. Both standard chemo- and radiotherapy were attempted. Unfortunately, the patient developed lung metastases six months after treatment completion. After obtaining written consent and approval from the Institutional Review Board of the Linkou Chang Gung Memorial Hospital, Taiwan (IRB approval numbers: 201801202B0 and 201801840B0), the following patient materials were collected: (1) primary uterine DEC specimens, (2) lung metastasis samples, and (3) peripheral blood aliquots. Formalin-fixed, paraffin-embedded (FFPE) samples of both primary DEC and lung metastases were archived for subsequent analyses. The patient died of disease 12 months after the initial diagnosis.

### 2.2. Patient-Derived Xenograft Model

All animal experiments conformed to the ethical guidelines set forth by the Department of Agriculture’s Animal Protection Law and were reviewed and approved by the Institutional Animal Care and Use Committee (IACUC) of the Linkou Chang Gung Memorial Hospital (approval number: 2018072601). The PDX model–termed PDX-mLung–was established as previously described [13] using lung metastasis specimens as source material for the xenograft tumor. After rinsing in phosphate buffered saline (PBS), fresh samples of lung metastases were immediately cut into small sections (2.5–3.0 mm) and implanted into the subcutaneous flank region of anesthetized mice. The xenograft tumor volume was regularly monitored three days per week and, once the average volume reached 100 mm^3^, mice were sacrificed. Excised tumors were sub-implanted into the next passage of mice. FFPE samples of PDX-mLung were archived for subsequent analyses.

### 2.3. Whole-Exome Sequencing

DNA for whole-exome sequencing (WES) was extracted from 10 μm-thick FFPE sections from (1) primary uterine DEC specimens, (2) lung metastasis samples, and (3) PDX-mLung using a commercially available kit (QIAamp DNA FFPE Tissue Kit; Qiagen, Hilden, Germany). The patient’s genomic DNA was isolated from peripheral blood aliquots using the QIAamp DNA Blood Midi Kit (Qiagen). The concentration and integrity of purified DNA were examined using the Quanti-iT dsDNA HS assay (Invitrogen, Carlsbad, CA, USA) and a fragment analyzer (Advanced Analytical Technologies, Ankeny, IA, USA), respectively [14]. Enrichment of the whole exome including flanking intronic regions was undertaken using the Twist Human Core Exome EF Multiplex Complete Kit (Twist Bioscience, South San Francisco, CA, USA). Briefly, genomic DNA (50 ng) was subjected to enzymatic fragmentation and the resulting sheared fragments were used for library construction according to the manufacturer’s instructions. Next-generation WES was carried out on a Novaseq 6000 System (Illumina, San Diego, CA, USA).

### 2.4. Identification of Single Nucleotide Variants and Insertion/Deletions in the Patient-Derived Xenograft Model 

All sequencing reads (fastq format) were trimmed to remove low quality and adaptor-containing sequences using trimmomatic [15]. After trimming, DNA from peripheral blood, primary DEC specimens, and lung metastatic lesions were aligned to human reference genomes (GRCh38p7) using BWA MEM [16]. The same tool was used to align PDX-derived reads to human-mouse fusion reference genomes (GRCh38p7 and GRCm38). PDX-derived reads aligned to mouse genome were further removed before variant calling following the procedure described by Callari et al. [17]. Reads alignment quality was assessed on the Qualimap 2 platform [18]. Somatic single nucleotide variants (SNVs) and the insertion/deletions (indels) variant calling was performed with the Mutect2 [19] and Strelka2 [20] tools using default parameters. Only variants that were called by both tools and characterized by a variant allele fraction (VAF) ≥ 0.05 and a tumor/normal read depth ≥ 20 was considered significant. Variants were annotated using ANNOVAR [21].

### 2.5. Analysis of Copy Number Variations

Analysis of copy number variations (CNVs) was performed on DNA amplicons obtained from (1) PDX-mLung, (2) primary DEC specimens, and (3) lung metastasis samples. Somatic copy number calling was conducted using the Sequenza [22] and CNVkit tools [23] with tumor-normal paired and aligned reads (BAM format).

### 2.6. In Vivo Pharmacological Treatment of Patient-Derived Xenograft Model

Based on the molecular alterations identified in the xenograft tumor, PDX-mLung mice were randomized into four groups (*n* = 5 per group) to receive the following drugs: (1) standard platinum-based drug paclitaxel (10 mg/kg given intraperitoneally one day per week), (2) the fibroblast growth factor receptor (FGFR) inhibitor lenvatinib alone (10 mg/kg given orally five days per week), (3) the cell cycle inhibitor palbociclib alone (100 mg/kg given orally five days per week), and (4) a combination of lenvatinib (5 mg/kg given orally five days per week) and palbociclib (50 mg/kg given orally five days per week). Treatment responses were assessed using tumor volume measured with animal ^18^F-fluorodeoxyglucose (^18^F-FDG) positron emission tomography (PET) imaging. Upon completion of the experimental program, tumors were excised for additional investigations.

### 2.7. Animal ^18^F-Fluorodeoxyglucose Positron Emission Tomography 

PDX-mLung mice were injected with ^18^F-FDG (3.7 MBq) into a tail vein. After 60 min, NanoScan PET (PET1225; Mediso, Budapest, Hungary) images were acquired (scan time duration: 10 min) from animals in the prone position. All imaging procedures were performed on anesthetized (2% isoflurane) mice placed near the center of the field of view. Reconstructed images (voxel size: 0.4 × 0.4 × 0.4 mm) were obtained using the Tera-Tomo 3D image algorithm (4 iterations and 6 subsets). The volume of interest adjacent to the tumor was adjusted on images obtained in the coronal view. The mean standardized uptake value (SUVmean) was used for determining tracer uptake. All images were analyzed on a PMOD platform (version 4.004; PMOD Technologies, Zürich, Switzerland).

### 2.8. Bulk Tumor RNA Sequencing and Differential Expression Gene (DEG) Analysis

After 11 days of treatment, we prepared PDX tumor and treated with palbociclib, lenvatinib or their combination, mice were sacrificed, and RNA was extracted from PDX-mLung tumors. We used the KAPA mRNA HyperPrep kit (Roche, Basel, Switzerland) to enrich RNA before sequencing, which was carried out on a Novaseq 6000 System (Illumina). RNA raw reads were aligned using STAR [24] to the human-mouse fusion reference genome (GRCh38.85 and GRCm38.100) to quantify transcripts. Reads aligned to the mouse genome were removed before downstream differential expression gene (DEG) analysis. The quality of global reads alignment was evaluated by Qualimap 2 tool [18] and DEG analysis was performed using the DESeq2 R package [25]. To avoid over-estimation of the log-transformed fold changes of transcript abundances, the posterior approximation for the generalized linear model was applied to reduce the variance for genes with limited information for statistical inference [26]. Gene ontology (GO) enrichment analysis was conducted using clusterProfiler [27] and NetworkAnalyst 3.0 [28].

### 2.9. Immunohistochemistry

IHC was performed as previously described [29]. Briefly, 4-μm-thick FFPE PDX-mLung, primary DEC, and lung metastasis specimens were deparaffinized in xylene and rehydrated through a series of graded ethanol washes in water. Heat-induced epitope retrieval was performed at 100 °C using citrate-based buffer (pH 6.0; BOND Epitope Retrieval Solution 1; Leica Biosystems, Nussloch, Germany) for FGFR2 or an EDTA-based buffer (pH 9.0; BOND Epitope Retrieval Solution 2, Leica Biosystems) for ER, PR, and cyclin E2 (CCNE2). Sections were immunostained with antibodies against ER (1:200 dilution; 6F11, Leica Biosystems), PR (1:500 dilution; PGR-312-L, Leica Biosystems), CCNE2 (1:200 dilution; 11935-1-AP, Proteintech, Rosemont, IL, USA), FGFR2 (1:100 dilution; SAB 32586, SAB, College Park, MD, USA), and p16 (1: 10 dilution; E6H4, ready for use, Ventana, Tucson, AZ, USA) using a BOND Polymer Refine Detection system (Leica Biosystems) on an automated IHC stainer. Definition of histoscore was the percentages of positive cells multiplied by their staining intensity.

### 2.10. Cell Culture

Human EC cells AN3CA were purchased from the American Type Culture Collection (Manassas, VA, USA). ARK1 and ARK2 cells were provided by Dr. Alessandro Santin (Yale University, School of Medicine, New Haven, CT, USA) [30]. The Ishikawa human endometrial cancer cell line was kindly obtained from Dr. Masato Nishida (Kasumigaura Medical Center, Ibaraki, Japan) [31]. AN3CA and Ishikawa cells were grown in α-minimum essential medium (α-MEM) containing 10% (V/V) FBS and 1% (V/V) penicillin/streptomycin. ARK1 and ARK2 cells were grown in RPMI-1640 medium containing 10% (V/V) FBS and 1% (v/v) penicillin/streptomycin.

### 2.11. Western Blot Analysis

The protocol used for western blot has been previously described in detail [29]. In brief, cells were harvested, washed twice with PBS, and lysed in ice-cold radioimmunoprecipitation assay (RIPA) lysis buffer. Lysates were boiled in 4× SDS-sample buffer and resolved on SDS-PAGE. SDS-PAGE-separated proteins were transferred by electrophoresis onto a nitrocellulose membrane. Finally, blots were probed with appropriate primary and appropriate secondary antibodies, as follows: p-Rb (9307; Cell Signaling Technology, Danvers, MA, USA), CCNE2 (11935-1-AP; Proteintech), p-ERK (SC-7383; Santa Cruz Biotechnology, Dallas, TX, USA), ERK (SC-154; Santa Cruz Biotechnology), FGFR2 (SC-6930; Santa Cruz Biotechnology), CDK2 (SC-6248; Santa Cruz Biotechnology), CDK4 (SC-23896; Santa Cruz Biotechnology), CDK6 (19117-l-AP; Proteintech), CDKN2A/p16 INK4a (ab16880; Abcam, Cambridge, UK) and β-actin (SC-47778; Santa Cruz Biotechnology). Densitometry readings/intensity ratio of each band. In addition, the whole blot showing all the bands with all molecular weight markers on the Western in the Supplementary Figure S5.

### 2.12. Cell Viability Assay

Cells (~1 × 10^4^) were seeded into each well of a 96-well culture plate for 24 h. After treatment with lenvatinib, palbociclib, or their combination, the MTT viability assay was performed by adding MTT (5 mg/mL, 25 μL) into each well. Following incubation at 37 °C for 4 h, the supernatant was discarded and DMSO (100 μL) was added to each well. After shaking to promote formazan dissolution, absorbance was read at 570 nm in a multiwell spectrophotometer (VICTOR 2; Perkin Elmer GMI, Ramsey, MN, USA) [29].

### 2.13. Reverse Transcription PCR

Total RNA was extracted using TOOLSmart RNA Extracter (BIOTOOLS CO. Ltd., New Taipei City, Taiwan) according to the manufacturer’s instructions. RNA samples were subjected to reverse transcription PCR (RT-PCR). PCR amplifications of p16 and GAPDH transcripts were performed in a reaction mixture (volume: 25 μL containing cDNA, forward and reverse primers (10 μmol each) specific for the p16 and GAPDH transcript, 2.5 μL of HotStart Taq PCR buffer with MgCl_2_ (10×), 1 μL of dNTP mixture (200 μM each), and one unit of taq DNA polymerase (Protech Systems Co., Ltd., Taipei, Taiwan). Primer sequences were: p16 F: 5’-GCCTTTTCACTGTGTTGGA-3’, p16 R: 5’- TATCATGAAGTCGACAGCTT-3’; GAPDH F: 5′-GGTATCGTGGAAGGACTCATGAC-3′, GAPDH R: 5′-ATGCCAGTGAGCTTCCCGT-3′. The PCR conditions were as follows: initial denaturation and hot start at 95 °C for 10 min; 30 cycles at 95 °C for 30 s, at 55 °C for 30 s, and at 72 °C for 30 s; final extension at 72 °C for 5 min. PCR products were visualized by 1.5% agarose gel electrophoresis followed by ethidium bromide staining. Two distinct bands were evident for p16 and GAPDH (113 bp and 188 bp, respectively).

### 2.14. Quantitative Real-Time PCR

The results of CNVs analysis for the *CCNE2* gene were validated by quantitative real-time PCR (QPCR) analysis for DNA expression [13]. The results were normalized to the expression levels of a housekeeping gene (*RAD51*). The primer sequences were as follows: CCNE2 F: 5′-ATTGGGATGTAACCCCATTG-3′, CCNE2 R: 5′-TCTTCACTGCAAGCACCATC-3′; RAD51 F: 5′-TACTACCTCAATGATGGGACC-3′, RAD51 R: 5′-AGGACTAATCTCCAAATAAACTAC-3′. Additionally, QPCR was used to investigate mRNA expression levels of both *CCNE2* and *FGFR2* in cell lines. The results were normalized to the expression levels of a housekeeping gene (*GAPDH*). The primer sequences were as follows: CCNE2 F: 5′-CCTCATTATTCATTGCTTCCAAAC-3′, CCNE2 R: 5′-TTCACTGCAAGCACCATCAG-3’; FGFR2 F: 5′-TTCTTGGAGCCTGCACAC-3′, FGFR2 R: 5’-CGGGCTCGGAGGTATTC-3′; GAPDH F: 5′-GGTATCGTGGAAGGACTCATGAC-3′, GAPDH R: 5′-ATGCCAGTGAGCTTCCCGT-3′. PCR products were sequenced and confirmed by Sanger sequencing.

### 2.15. Analysis of Synergy between Palbociclib and Lenvatinib

Analysis of synergy between lenvatinib and palbociclib was conducted by calculating the combination index (CI) using the CompuSyn software 1.0 (ComboSyn Inc., Paramus, NJ, USA); to this aim, the median effect principle of the Chou-Talalay method was applied [32]. A CI value close to 1 indicates an additive effect whereas a CI > 1 indicates an antagonistic effect. A CI value < 1 denotes a synergistic effect. The plotting of dose-effective curves for lenvatinib and palbociclib (given alone or in combination at different doses) was performed using the median effect equation (Fa/Fu = (D/Dm) m), where D indicates the given dose, Dm the dose required for 50% effect, Fa the fraction affected by D, Fu is the unaffected fraction (1 − Fa), and m the coefficient of sigmoid for the dose-effect curve. The synergy of lenvatinib and palbociclib was also examined in the MTT assays.

### 2.16. Statistical Analysis

Overall survival (OS) was defined as the interval from the date of diagnosis to the date of death from any cause, and censoring was performed on the date of the last follow-up (i.e., administrative censoring). Kaplan-Meier estimate curves were generated and survival differences were compared with the log-rank test. All analyses were carried out in SPSS, version 22.0 (IBM, Armonk, NY, USA). Statistical significance was determined by a two-tailed *p* value < 0.05.

## 3. Results

### 3.1. The Patient’s Derived Xenograft Model Retained the Original Histologic Characteristics of Human Dedifferentiated Endometrial Carcinoma

**Figure 1:**
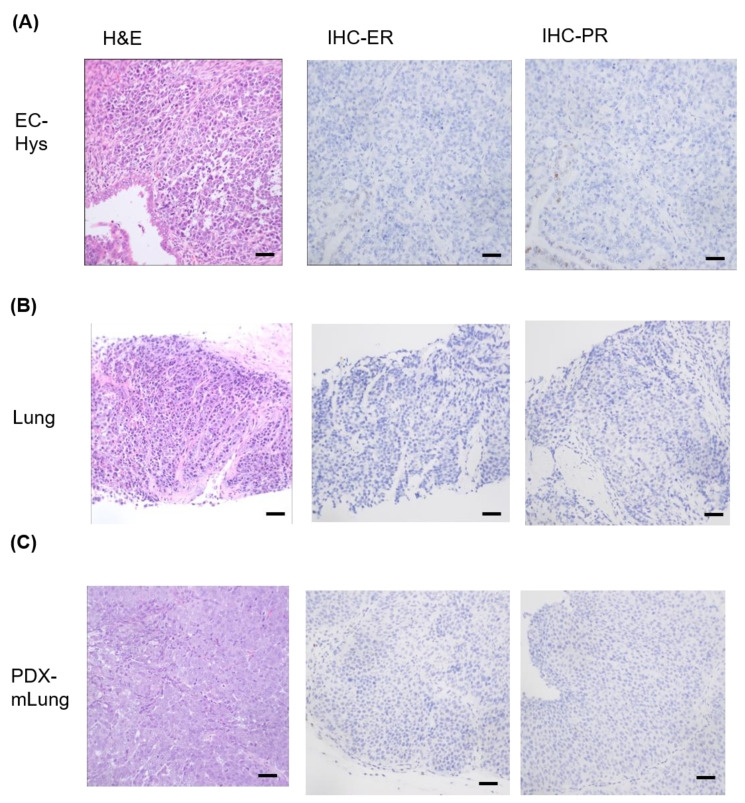
Representative histology images of primary dedifferentiated endometrial carcinoma (**A**) hysterectomy (hys) specimen; (**B**) lung metastasis; and (**C**) PDX-mLung. Hematoxylin and eosin (H&E) staining and results of immunohistochemistry (IHC) for estrogen receptor (ER) and progesterone receptor (PR) expression (Scale bar = 50 μm).

After failure of previous attempts to develop a model using primary uterine specimens, a patient-derived xenograft model of DEC–termed PDX-mLung–was successfully established over 3–4 weeks from lung metastatic lesions. Histologically, the PDX-mLung was characterized by a homogenous, non-cohesive sheets of monotonous tumor cells with large nuclei and a lack of glandular differentiation, resembling the undifferentiated carcinoma component of the endometrial tumor (Figure 1A) [4]. The parental tumor (lung metastasis) showed only the undifferentiated carcinoma component, whose key histologic characteristics were retained in PDX-mLung (Figure 1B,C). Both the parental patient’s lung metastatic lesions and PDX-mLung were consistently negative for both ER and PR immunohistochemical staining (Figure 1B,C).

### 3.2. The Patient’s Derived Xenograft Model Retained the Original Genetic Characteristics of Human Dedifferentiated Endometrial Carcinoma

**Figure 2:**
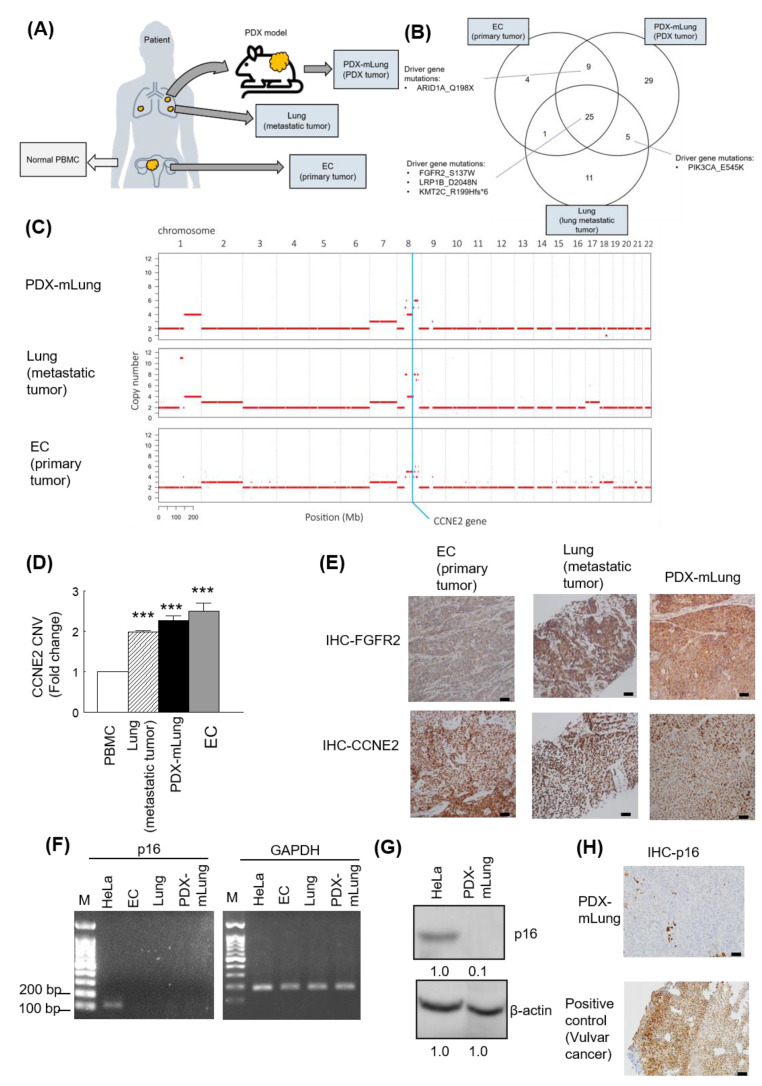
Experimental workflow for whole-exome sequencing (WES) in primary dedifferentiated endometrial carcinoma, lung metastasis, and PDX-mLung. (**A**) Schematic overview of sample collection. (**B**) Venn diagram of driver gene mutations identified in primary dedifferentiated endometrial carcinoma, lung metastasis, and PDX-mLung. (**C**) Comparison of copy number variations (CNVs) according to the results of WES. (**D**) Analysis of *CCNE2* CNVs (qPCR) in peripheral blood mononuclear cells, primary dedifferentiated endometrial carcinoma, lung metastasis, and PDX-mLung. (**E**) Results of immunohistochemistry for *FGFR2* and *CCNE2* expression in primary dedifferentiated endometrial carcinoma (left panel), lung metastasis (middle panel) and PDX-mLung (right panel). (**F**) Agarose gel electrophoresis of RT-PCR products (p16 and GAPDH genes). M: 100 bp DNA marker; the size of the nucleotide ladder is indicated on the left side (100 to 200 bp). GAPDH was used as internal control. (**G**) Protein expression of p16 in HeLa and PDX-mLung, respectively. β-actin served as control for normalization. The numbers below the data mean densitometry-derived values normalized to HeLal (set to 1). β-actin served as loading control for normalization (**H**) Results of immunohistochemistry for p16 in PDX (upper panel) and vulvar carcinoma. Vulvar carcinoma specimens expressing p16 were used as positive controls (lower panel; scale bar = 50 μm).

Using variant allele fractions estimates for known SNVs [33], the NGSCheckMate tool was used to assess the extent of genetic similarities between PDX-mLung, the patient’s lung metastatic lesions, and the primary DEC. The genetic identity of PDX-mLung with lung metastases and primary DEC were 98.4% and 97.7%, respectively. Comparisons were also undertaken in terms of WES and CNVs (Figure 2A). On analyzing WES data, PDX-mLung, lung metastatic lesions, and the primary DEC were found to share somatic mutations in the *FGFR2* (S137W), *LRP1B* (D2048N), and *KMT2C* (R199Hfs*6) genes (Figure 2B). The results of CNVs analysis consistently revealed the presence of *CCNE2* amplifications (Figure 2C), and the findings were successfully replicated by qPCR analysis using the patient’s DNA extracted from whole blood as control (Figure 2D). Given that both *FGFR2* mutations and *CCNE2* amplifications are potentially druggable, the protein expression of these two molecules was further analyzed with immunohistochemistry. The results revealed that both FGFR2 and CCNE2 were overexpressed in both PDX-mLung and the patient’s lung metastatic lesions (Figure 2E).

### 3.3. Pharmacological Targeting of the FGFR2 Mutation and CCNE2 Amplification with Lenvatinib and Palbociclib

**Figure 3:**
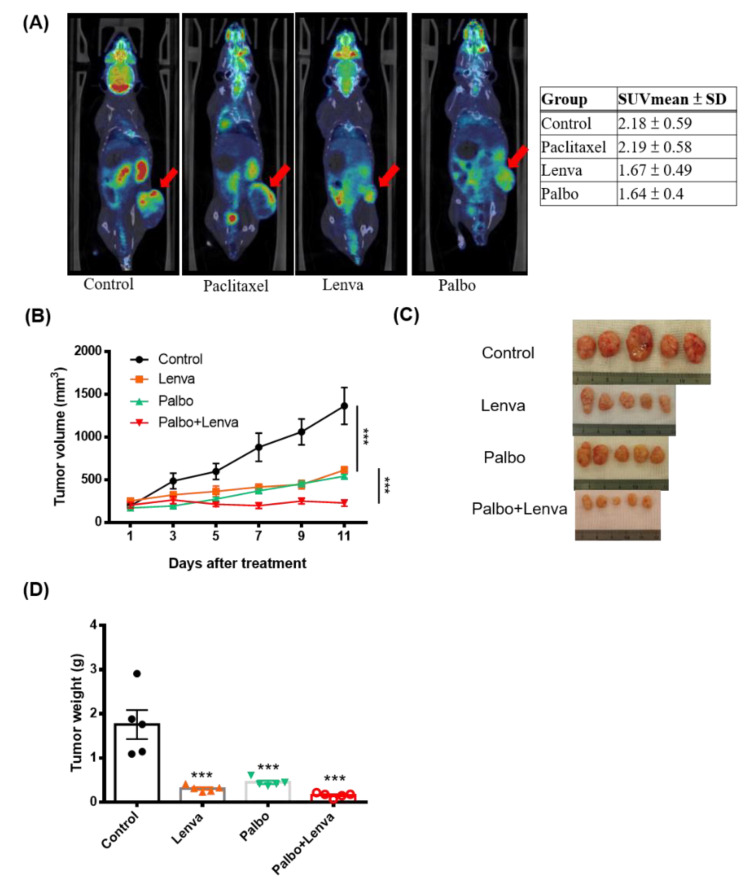
Identification of druggable molecular targets in PDX-mLung. PDX-mLung mice were randomized (*n* = 5 per group) to receive the fibroblast growth factor receptor inhibitor lenvatinib alone (10 mg/kg given orally five days per week), the cell cycle inhibitor palbociclib alone (100 mg/kg given orally five days per week), and a combination of lenvatinib (5 mg/kg given orally five days per week) and palbociclib (50 mg/kg given orally five days per week). Control animals were left untreated. (**A**) Whole-body ^18^F-FDG PET imaging was used to monitor tumor growth by calculating mean standardized uptake values (SUVmean), (**B**) tumor volume, (**C**) gross tumors, and (**D**) tumor weight. *** *p* < 0.0001.

The results of annotated variants (missense mutations and non-sense mutations) detected in three tumors are provided in Supplementary Table S1. Based on the PDX-mLung molecular profile, we first targeted the*FGFR2* (p.S137W) mutation with the FGFR inhibitor erdafitinib–an orally administered drug that has been previously used for advanced cancer [34]. However, PDX did not respond to erdafitinib (Supplementary Figure S1). Since the loss of p16 (CDKN2A) expression has been associated with sensitivity to the CDK4/6 inhibitor palbociclib in melanoma cell lines [35] and breast cancer patient [36], we further investigated p16 RNA and protein expression in metastatic lung lesion and PDX-mlung. Loss of p16 expression was observed on RT-PCR, western blot and immunohistochemistry analysis (Figure 2F,G,H). Starting from the observations of CCNE2 gain and p16 loss in PDX-mLung, we applied the cell cycle inhibitor palbociclib [37] and the multityrosine kinase inhibitor lenvatinib. The therapeutic effects of these targeted treatments were compared with those of standard paclitaxel-based adjuvant chemotherapy. The results revealed that both lenvatinib and palbociclib outperformed paclitaxel in terms of reduction of tumor volume (Figure 3 and Supplementary Figure S2). Similar in vivo findings were observed using animal ^18^F-FDG PET imaging (Figure 3A and Supplementary Figure S3).

### 3.4. Synergistic Therapeutic Effects of Lenvatinib and Palbociclib

We further examined whether lenvatinib and palbociclib were able to exert synergistic therapeutic effects in the PDX-mLung model. Compared to either lenvatinib (10 mg/kg) alone or palbociclib (100 mg/kg) alone, a combination of the two drugs (palbociclib 50 mg/kg and lenvatinib 5 mg/kg) exhibited synergistic effects (Figure 3B–D and Supplementary Table S2). Therefore, this synergy was further investigated in RNA-sequencing and EC cell lines.

### 3.5. RNA Sequencing Revealed Expression Differences in PDX-mLung Treated with a Combination of Palbociclib and Lenvatinib Versus Control

**Figure 4:**
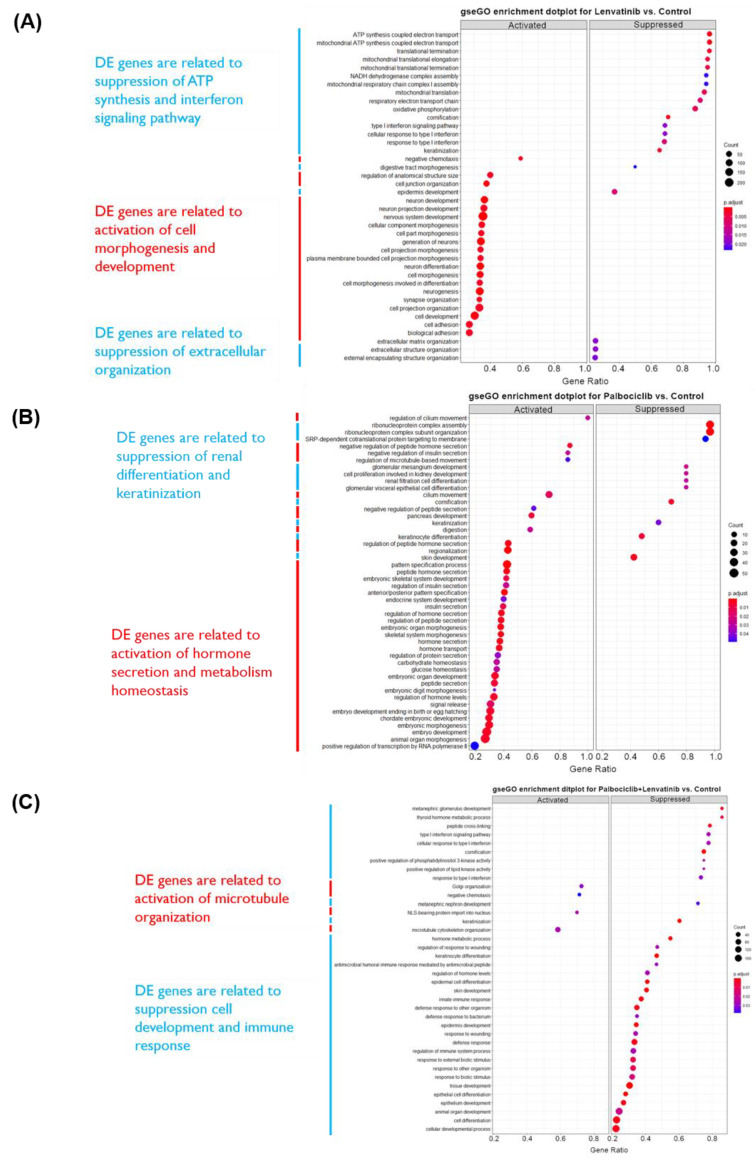
Differentially expressed genes of PDX-mLung treated with different drugs. (**A**) PDX-mLung treated with lenvatinib (**B**) PDX-mLung treated with palbociclib. (**C**) PDX-mLung treated with lenvatinib and palbociclib. DE: differential expression. Functional annotation analyses were performed to establish signaling pathways, biological processes and molecular functions associated with differentially expressed (DE) genes (left-hand side).

The effects of palbociclib and lenvatinib were assessed by means of RNA sequencing of PDX-mLung (mapping rate of each sample: 85–98% ratio of human reads; Supplementary Table S3). Differentially expressed genes are displayed using volcano plots (EnhancedVolcano R package; https://github.com/kevinblighe/EnhancedVolcano, accessed on 14 November 2021, Supplementary Figure S4). DEGs (log2 fold change ≥ 1 and adjusted *p* value < 0.05) were subjected to functional annotation through the GO enrichment tool of clusterProfiler (R package). Signature-enriched pathways following lenvatinib treatment included the suppression of ATP synthesis, interferon (IFN) signaling, and extracellular organization pathways. Raw gene counts for all tested tumor samples are summarized in Supplementary Table S4. Signature-enriched pathways following palbociclib included activation of hormone secretion and metabolism homeostasis (Figure 4). Functional annotation analysis was subsequently performed using the Annotation, Visualization, and Integrated Discovery (DAVID) (https://david-d.ncifcrf.gov/, accessed on 28 September 2021) dataset. Specifically, we sought to identify genes that showed a log2 downregulation higher than 1.5 folds following exposure to lenvatinib and palbociclib (Supplementary Tables S5). Differentially expressed genes were further examined using KEGG. The results revealed that genes in the MAPK pathway and gap junction pathways were the most commonly affected by lenvatinib and palbociclib (*p* value = 3.4 × 10^−3^ and 4.9 × 10^−3^, respectively.

### 3.6. Lenvatinib and Palbociclib Synergistically Inhibited Cell Viability and Decreased CCNE2 Expression in Endometrial Cancer Cell Lines

**Figure 5:**
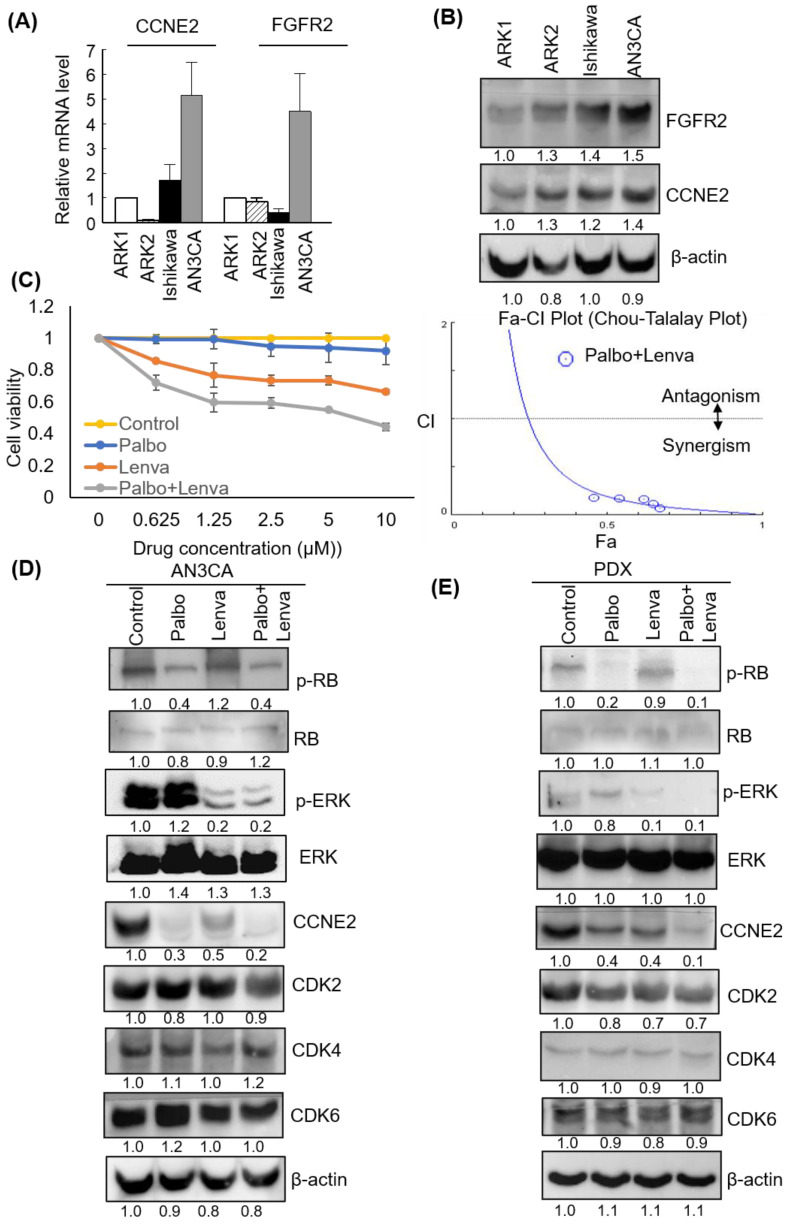
Palbociclib and lenvatinib synergistically inhibited *CCNE2* expression levels and decreased viability of endometrial cancer (EC) cells. *CCNE2* and *FGFR2* (**A**) mRNA and (**B**) protein expression in ARK1, ARK2, Ishikawa, and AN3CA cell lines, respectively. β-actin served as control for normalization. The numbers below the data mean densitometry-derived values normalized to ARK1 (set to 1). β-actin served as loading control for normalization. (**C**) AN3CA cells were treated for 48 h with vehicle (−) or different doses of palbociclib alone (0.625, 1.25, 2.5, 5, and 10 μM), lenvatinib alone (0.625, 1.25, 2.5, 5, and 10 μM), or their combination. Cell survival was analyzed with the MTT assay. Data from three different experiments (each performed in triplicate) are expressed as fold changes ± standard deviations relative to vehicle-treated cells (left panel). The synergistic effects of palbociclib and lenvatinib were analyzed using the CompuSyn software (right. panel). (**D**) Palbociclib (10 μM), lenvatinib (10 μM) and their combination were used to treat AN3CA cells for 48 h. (**E**) Tissues from the PDX-mlung model treated with palbociclib, lenvatinib, or their combination were collected. Cell lysates were subsequently resolved on SDS-PAGE and subjected to immunoblotting with antibodies raised against p-RB, RB, p-ERK1/2, ERK1/2, CCNE2, CDK2, CDK4, CDK6, and β-actin. The numbers below the data mean densitometry-derived values normalized to control (set to 1). β-actin served as loading control for normalization. Abbreviations: Palbo, palbociclib; Lenva, lenvatinib; Palbo+Lenva: palbociclib and lenvatinib given in combination.

*FGFR2* and *CCNE2* expression levels were investigated by qPCR and western blot in different EC cell lines–including ARK1, ARK2, Ishikawa, and AN3CA cells. The results revealed that–across all of the examined EC cell lines–AN3CA cells had the highest mRNA and protein FGFR2 and CCNE2 expression (Figure 5A,B). The MTT assay and Chou-Talalay plot analysis showed that lenvatinib and palbociclib synergistically inhibited the viability of AN3CA cells (Figure 5C). Moreover, western blot experiments revealed that lenvatinib and palbociclib were capable of suppressing ERK1/2 and RB phosphorylation, respectively resulting in downstream regulation of *CCNE2* (Figure 5D). Similar findings were observed in the PDX-mLung model (Figure 5E). Collectively, these data indicate that lenvatinib and palbociclib synergistically inhibited CCNE2 expression via ERK1/2 and RB signaling. Notably, these findings were consistent with the data on the MAPK pathway observed in KEGG analysis after lenvatinib treatment.

### 3.7. Increased CCNE2 Copy Number Variations and mRNA Expression Levels Portended a Poor Overall Survival in Patients with Endometrial Cancer

**Figure 6:**
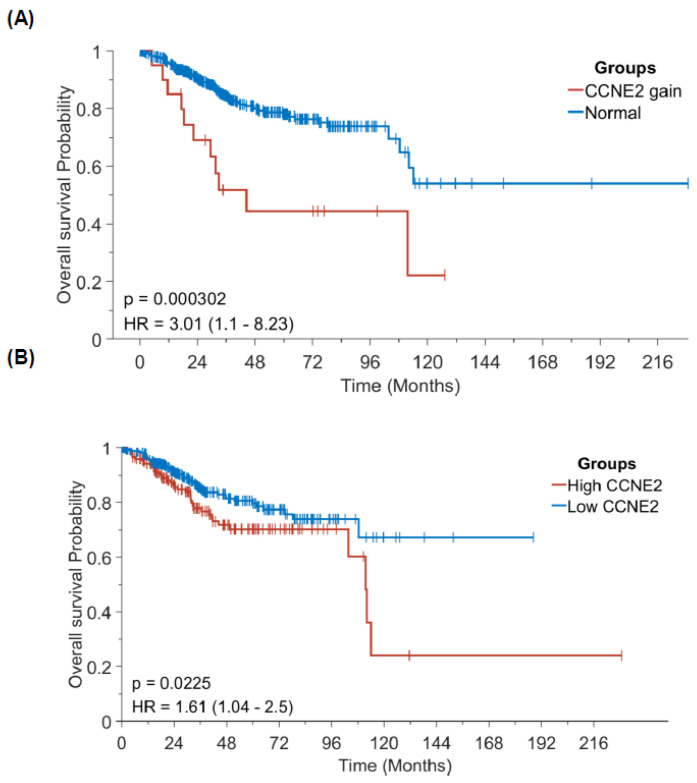
Prognostic significance of *CCNE2* copy number variations and mRNA expression in patients with endometrial cancer (data source: Cancer Genome Atlas-Uterine Corpus Endometrial Carcinoma). Kaplan-Meier plots of overall survival according to *CCNE2* (**A**) copy number variations and (**B**) mRNA expression.

Finally, we investigated the prognostic impact of *CCNE2* CNVs and mRNA expression levels using patient data obtained from the Cancer Genome Atlas-Uterine Corpus Endometrial Carcinoma (TCGA-UCEC) database. After dichotomization of patients with EC according to optimal cutoff for *CCNE2* copy number variations (>2 *versus* ≤2; Figure 6A), we found that cases with >2CNVs of CCNE2 had a significantly lower OS (*p* < 0.0001). On analyzing the prognostic effect of *CCNE2* mRNA expression levels (cutoff: 0.68), patients with an increased expression (n = 185) were characterized by a less favorable OS (n = 347; *p* = 0.0225; Figure 6B). 

## 4. Discussion

Human DEC is characterized by a mixture of different histologic components (low-grade EC mixed with undifferentiated carcinoma components) [6] that may pose diagnostic challenges and reflects a highly aggressive biological nature. In our study, PDX-mLung was developed over a 4-week period, consistent with the 2–11-week period previously reported for other PDX models of high-risk endometrial malignancies [11,12]. There are three principal findings from this study. First, we were able to successfully establish a murine model–termed PDX-mLung–which was found to retain the original characteristics of human DEC both in terms of histology and molecular alterations. Second, extensive molecular investigations revealed that PDX-mLung and, consequently, human DEC, exhibited druggable alterations–including a *FGFR2* mutation and *CCNE2* amplification. In a preclinical study conducted in PDX-mLung, the former was targeted with the FGFR inhibitor lenvatinib while the latter with the cell cycle inhibitor palbociclib. The combination of the two drugs was found to exhibit synergistic therapeutic effects against in vivo tumor growth. Finally, using patient data obtained from the TCGA-UCEC database, we demonstrated that both high *CCNE2* CNVs and mRNA expression levels were associated with a less favorable OS in patients with EC.

In this study, we found that DEC had two main druggable molecular alterations, (i.e., a *FGFR2* mutation targeted by lenvatinib and *CCNE2* amplification targeted by palbociclib). Consequently, PDX-mLung was used as a preclinical model to test the efficacy of both drugs, given either alone or in combination. The significant role played by the IFN pathway in response to lenvatinib treatment [38] supports the clinical utility of lenvatinib in PDX-mLung. Moreover, activation of hormonal homeostasis following palbociclib treatment is consistent with our previous findings showing that palbociclib was capable of sensitizing endometrial cancer cells to megestrol acetate [29]. The question as to whether hormonal therapy may be used in combination with this drug in human DEC should be further investigated.

Interestingly, lenvatinib has been previously reported to have a potential anti-tumor activity in recurrent or metastatic EC [39,40]. Growing evidence indicates that dysregulation of the FGFR signaling pathway is involved in human tumorigenesis [41,42,43]. Similarly, an aberrant cell cycle control regulation is a key hallmark of malignant cells and CCNE2 plays a paramount role in the G1/S transition [44]. Studies have shown that CCNE2 overexpression is common in human malignancies and may portend adverse outcomes [45,46]. Notably, this finding was successfully replicated in our study on analyzing data of patients with EC included in the TCGA-UCEC database. Mechanistically, palbociclib can reduce CCNE2 expression by decreasing levels of phosphorylated RB [47,48] as confirmed by our findings in AN3CA cells that harbored FGFR2 mutation.

The concomitant presence of two key molecular alterations that drive human tumorigenesis (i.e., a *FGFR2* mutation and *CCNE2* amplification) may explain the biological aggressiveness of DEC. Moreover, lenvatinib is already known to be effective in endometrial carcinoma and approved in combination with immunotherapy [38]. While resistance to palbociclib may occur leading to increased cell cycle and growth factor in cancer cells [49], aggressive tumors such as DEC were treated with combined regimen of lenvatinib plus palbociclib to overcome molecular alternations of resistance to palbociclib alone. Interestingly, we found that lenvatinib and palbociclib given in combination provided synergistic effects against tumor growth in the PDX-mLung model. The combination also appeared to be helpful in magnifying the antiproliferative effects of each drug alone in AN3CA cells. Although these findings are preliminary, they provide proof-of-concept evidence that PDX models are useful to inform pharmacological treatment targeted on specific molecular alterations in highly aggressive human malignancies characterized by poor response to traditional therapies. Nonetheless, one of the major obstacles for their widespread implementation is the time-consuming experimental setup. Future clinical trials will be necessary to examine the optimal dosages and the utility of lenvatinib and palbociclib in women with DEC, as well as investigate whether *FGFR2* mutations and *CCNE2* amplifications may serve as markers of treatment response.

## 5. Conclusions

The results of our study provide proof-of-concept evidence on the value of PDX models for preclinical testing of molecularly informed drug therapy in difficult-to-treat human malignancies. Further clinical research is needed to examine more rigorously the potential usefulness of the lenvatinib and palbociclib combination in patients with DEC.

## Data Availability

The data presented in this study are available in supplementary material.

## Supplementary Materials

The following are available online at https://www.mdpi.com/article/10.3390/cancers13235962/s1, Figure S1: Tumor volume analysis after treatment according to druggable molecular targets in PDX-mLung. PDX-mLung mice were randomized (*n* = 2 per group) to receive standard platinum-based drug paclitaxel (10 mg/kg given intraperitoneally one day per week) or the fibroblast growth factor receptor inhibitor erdafitinib (15 mg/kg given orally five days per week); Figure S2: Identification of druggable molecular targets in PDX-mLung. PDX-mLung mice were randomized (*n* = 5 per group) to receive standard platinum-based drug paclitaxel (10 mg/kg given intraperitoneally one day per week), the fibroblast growth factor receptor inhibitor lenvatinib alone (10 mg/kg given orally five days per week), or the cell cycle inhibitor palbociclib alone (100 mg/kg given orally five days per week). (A) Tumor volume, (B) gross tumors, and (C) tumor weight changes observed in experimental mice. *** *p* < 0.0001. Figure S3: In vivo drug responses were observed using animal ^18^F-FDG PET imaging. (A) Whole-body ^18^F-FDG PET imaging was used to monitor tumor growth (red arrow) by calculating mean standardized uptake values (SUVmean) (B) Data shown represent mean ± standard error and were derived from representative PET Imaging of PDX-mLung mice (*n* = 2 per group). Lena: lenvatinib; Palbo: Palbociclib. Figure S4: Differentially expressed genes of PDX-mLung treated with different drugs. (A) PDX-mLung treated with lenvatinib. Total number of significant differentially expressed (DE) genes are 1342 with log2 fold change (FC) ≥ 1 and adjusted *p* value < 0.05. (B) PDX-mLung treated with palbociclib. Total number of significant DE genes: 1384 with log2 FC ≥ 1.5 and adjusted p value < 0.05. (C) PDX-mLung treated with lenvatinib and palbociclib. Total number of significant DE genes: 1178 with log2 FC ≥ 1 and adjusted *p* value < 0.05. Raw gene count of all tumor samples is provided in Supplementary Table S3. Figure S5: The whole blot showing all the bands with all molecular weight markers on the Western. Table S1: Mutation detected in PDX-mLung, lung (metastatic lesion) and EC (primary lesion) tumors; Table S2: Sequencing metrics in PDX-mLung tumor treated with lenvatinib, palbociclib or a combination of lenvatinib and palbociclib; Table S3: Gene expression in PDX-mLung tumor treated with lenvatinib, palbociclib or a combination of lenvatinib and palbociclib; Table S4. Functional Annotation Chart of PDX-mLung in response to drug treatment.
