# Supplementary material for: A Patient-Derived Xenograft Model of Dedifferentiated Endometrial Carcinoma: A Proof-of-Concept Study for the Identification of New Molecularly Informed Treatment Approaches
Source: Cancers (Basel). 2021 Nov 26;13(23):5962. doi: 10.3390/cancers13235962 (PMC8656552; doi:10.3390/cancers13235962)
Supplement: Supplementary file 1 [file cancers-13-05962-s001.zip › PDX_SUPPLEMENT_11132021.pdf]

A Patient-Derived Xenograft Model of Dedifferentiated Endometrial Carcinoma: A Proof-Of-Concept Study for the Identification of New Molecularly Informed Treatment Approaches

Chiao-Yun Lin, Ren-Chin Wu, Chen-Yang Huang, Chyong-Huey Lai, An-Shine Chao, Hsin-Pai Li, Chia-Lung Tsai, Elizabeth Joo-Wen Kuek, Cheng-Lung Hsu, Angel Chao

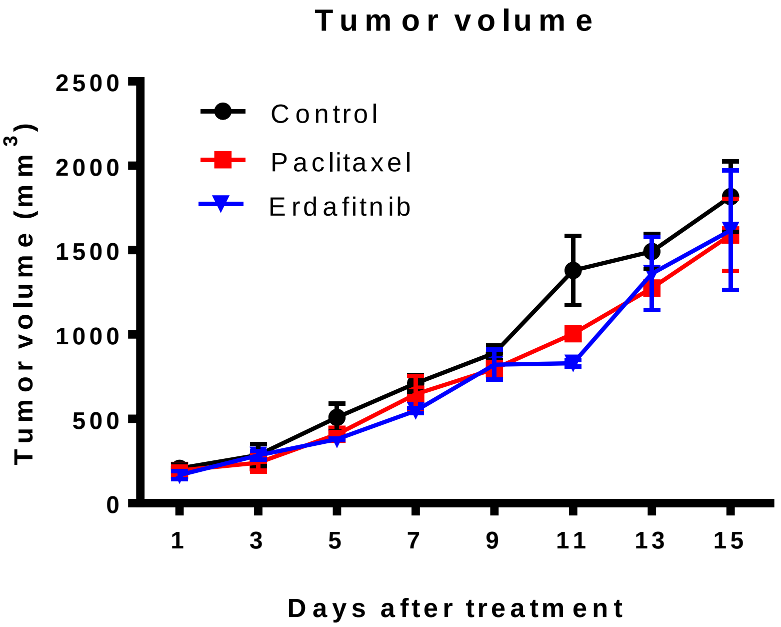

Figure S1. Tumor volume analysis of druggable molecular target in PDX-mLung. PDX-mLung mice were randomized (n = 2 per group) to receive standard platinum-based drug paclitaxel (10 mg/kg given intraperitoneally one day per week), and the fibroblast growth factor receptor inhibitor erdafitinib (15 mg/kg given orally five days per week).

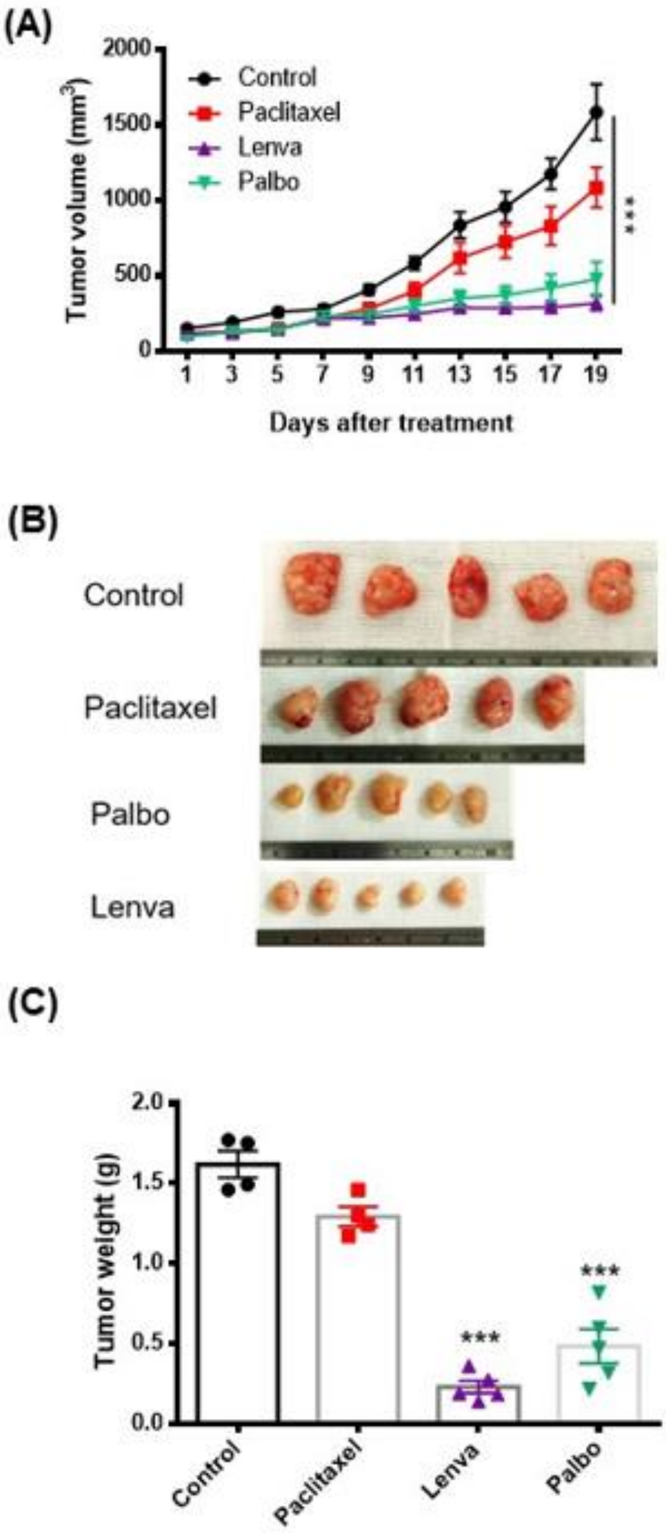

Figure S2. Identification of druggable molecular targets in PDX-mLung. PDX-mLung mice were randomized (n = 5 per group) to receive standard platinum-based drug paclitaxel (10 mg/kg given intraperitoneally one day per week), the fibroblast growth factor receptor inhibitor lenvatinib alone (10 mg/kg given orally five days per week), and the cell cycle inhibitor palbociclib alone (100 mg/kg given orally five days per week). **(A)** Tumor volume, **(B)** gross tumors, and **(C)** tumor weight changes of mice *** $p < 0.0001$.

(A)

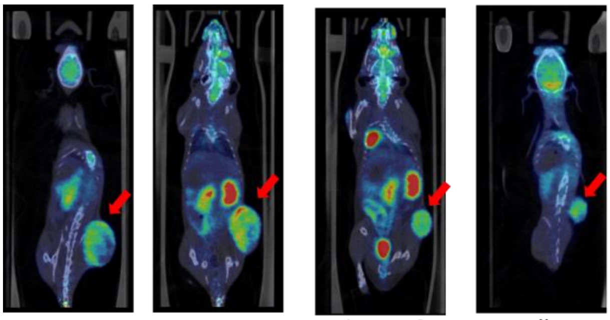

Control-2 Paclitaxel-2 Lenva-2 Palbo-2

Group	SUVmean ± SD
Control-2	1.74 ± 0.42
Paclitaxel-2	2.09 ± 0.55
Lenva-2	1.39 ± 0.37
Palbo-2	1.24 ± 0.27

(B)

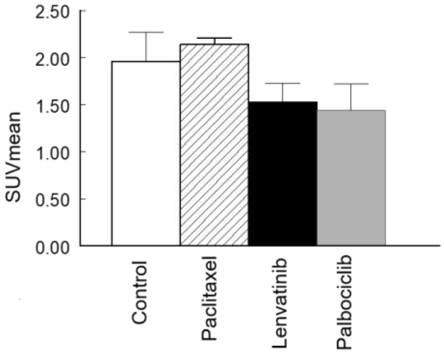

Figure S3. In vivo drug responses were observed using animal ^{18}F-FDG PET imaging. **(A)** Whole-body ^{18}F-FDG PET imaging was used to monitor tumor growth (red arrow) by calculating mean standardized uptake values (SUVmean) **(B)** Data shown represent mean ± standard error and were derived from representative PET Imaging of PDX-mLung mice (*n* = 2 per group). Lena: lenvatinib; Palbo: Palbociclib.

(A)

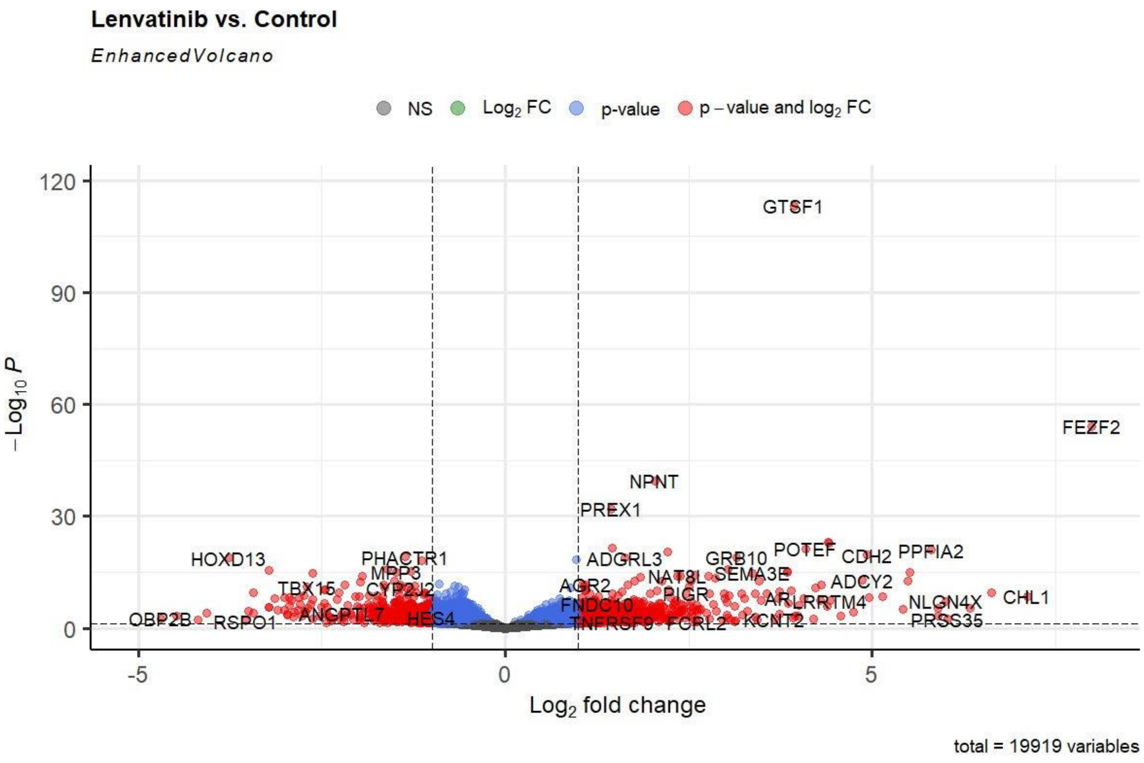
Lenvatinib vs. Control
EnhancedVolcano
NS Log2 FC p-value p – value and log2 FC
120
90
60
30
0
−Log10 P
-5
0
5
Log2 fold change
total = 19919 variables
GTSF1
FEZF2
NPNT
PREX1
HOXD13
PHACTR1
MPP3
TBX15
CYP2J2
ANGPTL7
OBP2B
RSPO1
HES4
ADGRL3
GRB10
POTEF
CDH2
PPFIA2
NAT8L
SEMA3E
AGR2
ADCY2
PIGR
FNDC10
AR
LRRTM4
NLGN4X
CHL1
TNFRSF9
FCRL2
KCNT2
PRSS35

(B)

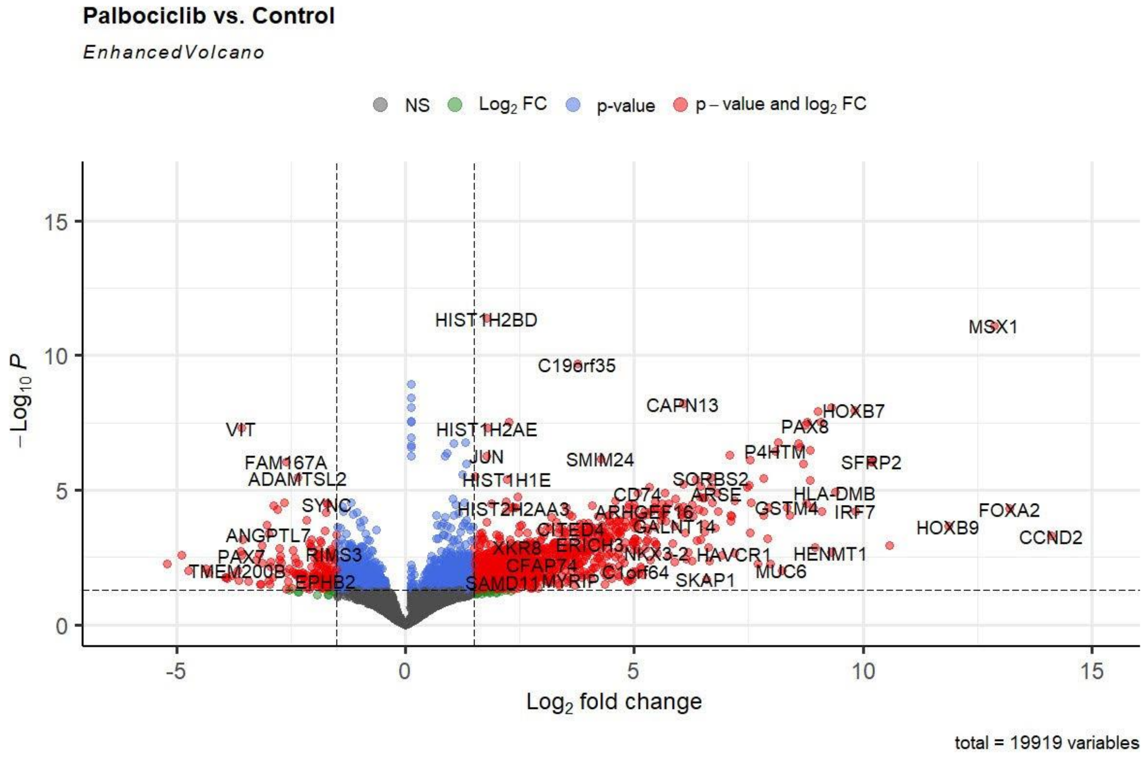
Palbociclib vs. Control
EnhancedVolcano
NS Log2 FC p-value p – value and log2 FC
15
10
5
0
−Log10 P
-5
0
5
10
15
Log2 fold change
total = 19919 variables
HIST1H2BD
MSX1
C19orf35
CAPN13
HOXB7
VIT
HIST1H2AE
PAX8
P4HTM
JUN
SMIM24
SFRP2
FAM167A
ADAMTSL2
HIST1H1E
SORBS2
CD74
ARSE
HLA-DMB
SYNC
HIST2H2AA3
ARHGEF16
GSTM4
IRF7
FOXA2
CITED4
GALNT14
HOXB9
CCND2
ANGPTL7
RIMS3
XKR8
ERICH3
NKX3-2
HAVCR1
HENMT1
PAX7
TMEM200B
CFAP74
C1orf64
MUC6
EPHB2
SAMD11
MYRIP
SKAP1

(C)

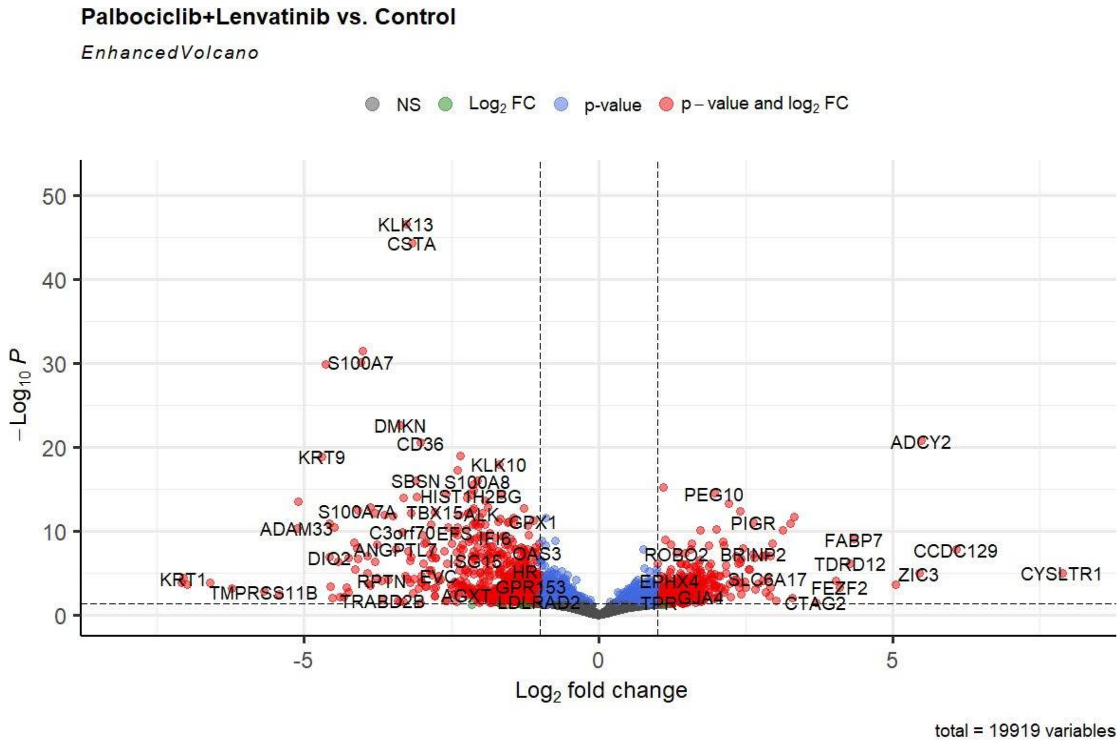

Figure S4. Differentially expressed genes of PDX-mLung treated with different drugs. **(A)** PDX-mLung treated with lenvatinib. Total number of significant differentially expressed (DE) genes are 1342 with log2 fold change (FC) ≥ 1 and adjusted *p* value < 0.05. **(B)** PDX-mLung treated with palbociclib. Total number of significant DE genes: 1384 with log2 FC ≥ 1.5 and adjusted p value < 0.05. **(C)** PDX-mLung treated with lenvatinib and palbociclib. Total number of significant DE genes: 1178 with log2 FC ≥ 1 and adjusted *p* value < 0.05. Raw gene count of all tumor samples is provided in Supplementary Table S3.

Figure 2G

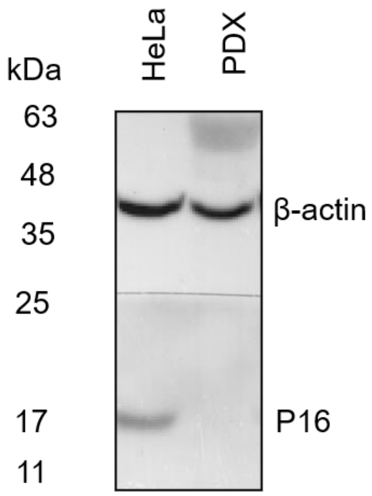

Figure 5B

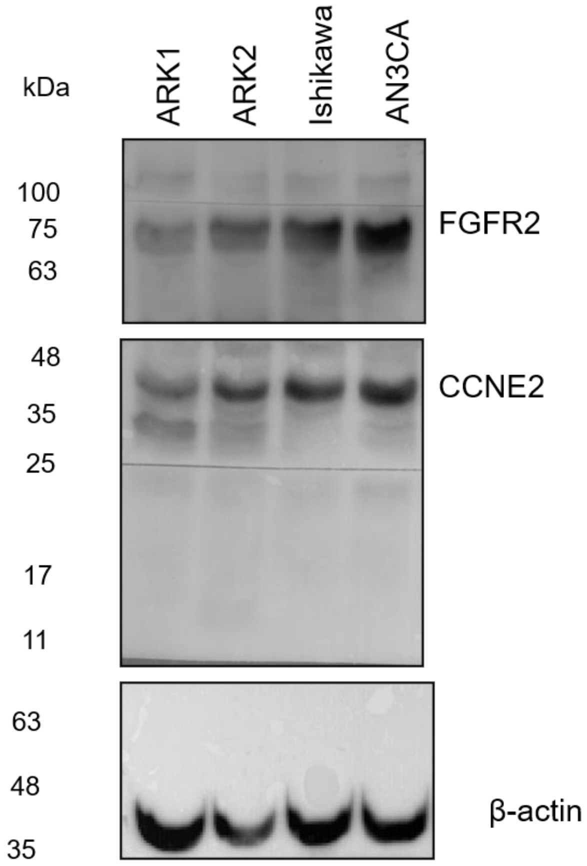

Figure 5D

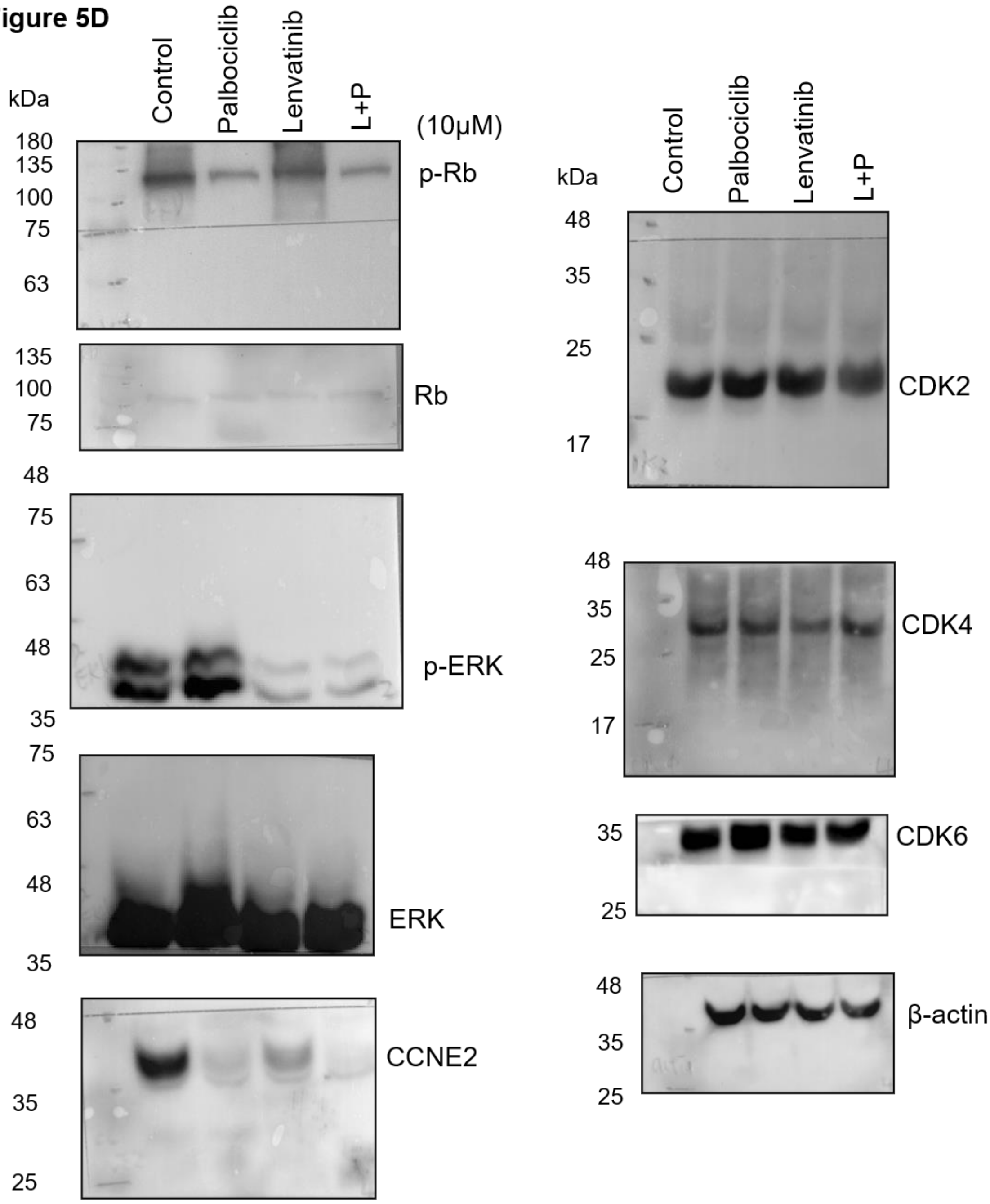
kDa
Control
Palbociclib
Lenvatinib
L+P
(10μM)
180
135
100
75
63
p-Rb
135
100
75
Rb
48
75
63
48
35
p-ERK
75
63
48
35
ERK
48
35
25
CCNE2
kDa
Control
Palbociclib
Lenvatinib
L+P
48
35
25
17
CDK2
48
35
25
17
CDK4
35
25
CDK6
48
35
25
β-actin

Figure 5E

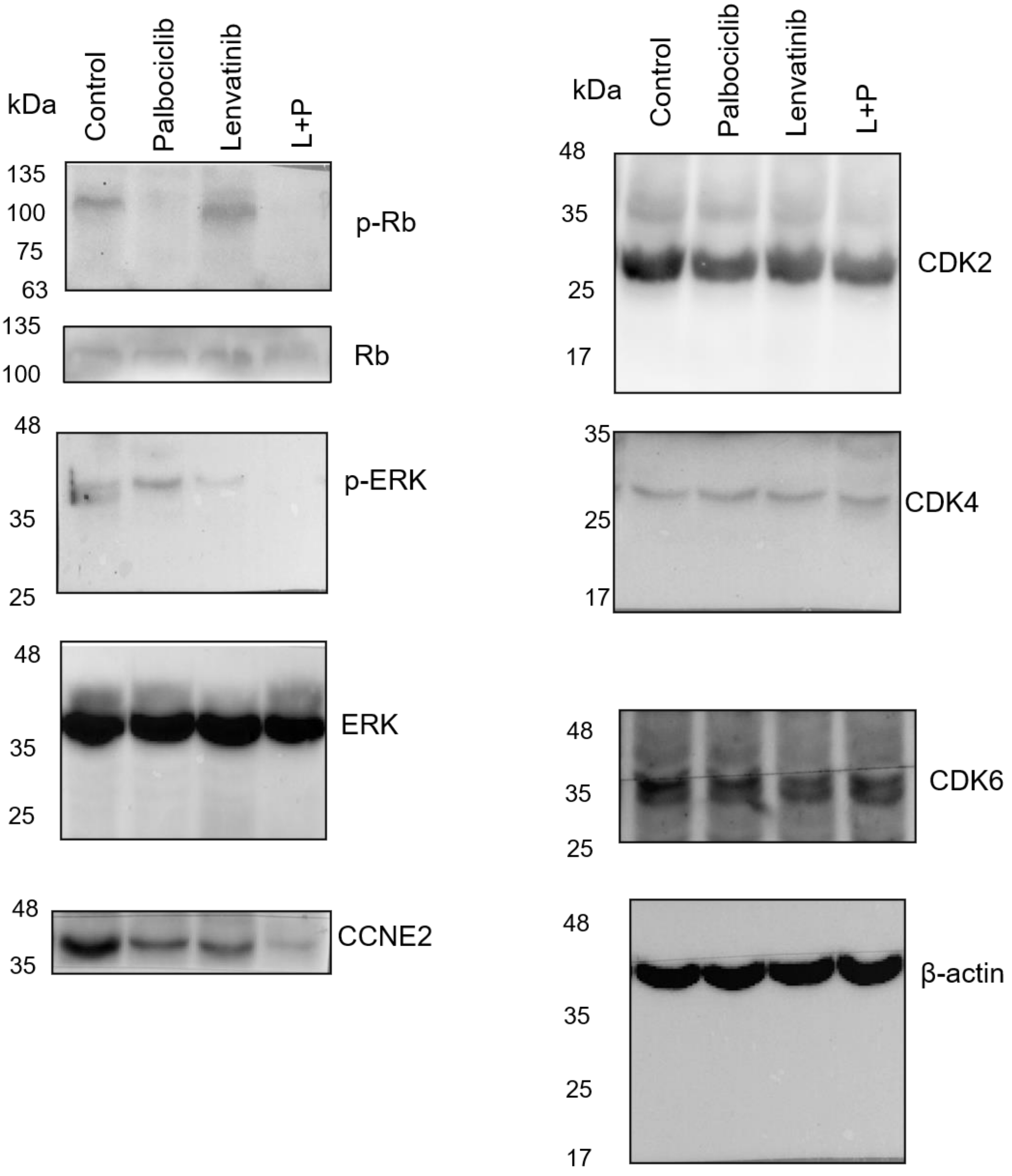

Figure S5. The whole blot showing all the bands with all molecular weight markers on the Western as shown above.
